# Hierarchical Surface Texturing of Hydroxyapatite Ceramics: Influence on the Adhesive Bonding Strength of Polymeric Polycaprolactone

**DOI:** 10.3390/jfb11040073

**Published:** 2020-10-03

**Authors:** Jonas Biggemann, Philipp Müller, David Köllner, Swantje Simon, Patrizia Hoffmann, Paula Heik, Jung Heon Lee, Tobias Fey

**Affiliations:** 1 Department of Materials Science (Institute of Glass and Ceramics), University of Erlangen-Nuernberg, Martensstr. 5, D-91058 Erlangen, Germany; Philipp-Lorenz-Mueller@web.de (P.M.); David.Koellner@fau.de (D.K.); Swantje.Simon@fau.de (S.S.); Patrizia.Hoffmann@fau.de (P.H.); Paula.Heik@fau.de (P.H.); 2 School of Advanced Materials Science & Engineering, Sungkyunkwan University (SKKU), Suwon 16149, Korea; JHLee7@skku.edu; 3 Frontier Research Institute for Materials Science, Nagoya Institute of Technology, Gokiso-cho, Showa-ku, Nagoya 466-8555, Japan

**Keywords:** hierarchical surface texturing, surface functionalization, hydroxyapatite, polycaprolactone, acid etching, tartaric acid, silane, adhesive bonding strength

## Abstract

The tailored manipulation of ceramic surfaces gained recent interest to optimize the performance and lifetime of composite materials used as implants. In this work, a hierarchical surface texturing of hydroxyapatite (HAp) ceramics was developed to improve the poor adhesive bonding strength in hydroxyapatite and polycaprolactone (HAp/PCL) composites. Four different types of periodic surface morphologies (grooves, cylindric pits, linear waves and Gaussian hills) were realized by a ceramic micro-transfer molding technique in the submillimeter range. A subsequent surface roughening and functionalization on a micron to nanometer scale was obtained by two different etchings with hydrochloric and tartaric acid. An ensuing silane coupling with 3-aminopropyltriethoxysilane (APTES) enhanced the chemical adhesion between the HAp surface and PCL on the nanometer scale by the formation of dipole–dipole interactions and covalent bonds. The adhesive bonding strengths of the individual and combined surface texturings were investigated by performing single-lap compressive shear tests. All individual texturing types (macro, micro and nano) showed significantly improved HAp/PCL interface strengths compared to the non-textured HAp reference, based on an enhanced mechanical, physical and chemical adhesion. The independent effect mechanisms allow the deliberately hierarchical combination of all texturing types without negative influences. The hierarchical surface-textured HAp showed a 6.5 times higher adhesive bonding strength (7.7 ± 1.5 MPa) than the non-textured reference, proving that surface texturing is an attractive method to optimize the component adhesion in composites for potential medical implants.

## 1. Introduction

A great variety of material properties are predominantly influenced by their surface characteristics such as the surface topography, morphology, roughness, energy, specific surface area, chemistry and wettability [1,2,3,4,5,6]. For biomaterials in particular, the surface represents the interface between living tissue and artificial implant and therefore governs the cascade of biological events after implantation [4,6]. This includes the initial adsorption of proteins and the subsequent attachment, proliferation and differentiation of cells on the implant [2,5,7,8]. The surface therefore determines the biomechanical fixation in bone (osseointegration) and thus the success and durability of the implant [2,5,7,8,9,10]. For ceramic implants, the surface, in addition, strongly affects the mechanical strength and wear resistance. Based on their inherent brittle nature, the strength of ceramics is highly sensitive to the existence of surface flaws (cracks, pores, scratches) and even in their absence failure may occur initiated from the microscopic roughness of large grains [11,12]. For that reason, commercial full-ceramic ZrO_2_ or Al_2_O_3_ artificial hip and knee implants are typically polished (R_a_ < 10 nm) to avoid surface defects and to achieve a high strength and a low-friction surface [13,14].

Surface texturing gained, however, great interest to manipulate the surface characteristics of implants in order to improve their performance and lifetime [13,15,16,17,18,19,20,21]. The introduction of periodic micro-textures (such as pits or bioinspired patterns) on hip implant surfaces not only reduced the contact area and thus the wear compared to the polished standard, but additionally provided an intrinsic lubrication and self-cleaning ability of emerging wear debris [13,15,16]. Surface modifications of bone implants, especially a surface roughening, were shown to enhance the cell adhesion, cell proliferation and promote bone ingrowth [7,8,9,22]. Moreover, ceramic surface modifications in dental restorative composites led to an improved component adhesion based on an increased contact surface, mechanical interlocking effects and activated functional groups at the surface for an enhanced chemical adhesion [17,18,19,20,21]. Hybrid ceramic–polymer composites are one of the most promising candidates as bone substitute materials, because of their capability to mimic the properties of bone by combining the low stiffness and damage tolerance of polymers with the high stiffness and strength of ceramics [23,24]. Of particular interest are implants consisting of bioactive ceramics such as the osteoconductive hydroxyapatite and bioresorbable polymers such as polycaprolactone, which are both approved for medical applications by the Food and Drug Administration (FDA) [25,26,27,28,29,30]. A remaining obstacle for the use as implants is the poor interface strength between both materials [26,28,31], which might be improved by applying a surface texturing of the hydroxyapatite (HAp) ceramic.

The currently established surface texturing techniques for ceramics can be classified according to their application type (additive, subtractive), the degree of order (random surfaces or well-ordered periodic patterns) and the dimensional scale of the generated surface texture. Well-ordered surfaces with strict periodic patterns (pits, protrusions or grooves) can be obtained on a nanometer to submillimeter scale by laser ablation [13,15,16], lithographic micro-molding [32,33] and additive manufacturing [1]. Grinding [7,20], etching [9,17,20], particle abrasion [9,17,18,20] and sol–gel coating processes [34,35] generate random surface patterns on a nanometer to micron scale. A subsequent silanization allows one to tailor the surface chemistry (surface energy and wettability) on an atomic scale [17,18,36]. A hierarchical surface texturing of hydroxyapatite ceramics is of particular interest to improve the in vivo biomechanical integration of a full ceramic implant as well as establishing a strong interface strength in HAp/polycaprolactone (PCL) composites but has so far never been investigated.

In this work, we therefore present the fabrication of hierarchical surface-textured HAp ceramics. Four types of computer-aided designed, periodic macro-surface textures (sub-millimeter scale) were manufactured by utilizing a micro-transfer molding technique published earlier [37,38,39]. A random surface roughening was introduced on a micron to nanometer scale by a subsequent acid etching. Additionally, samples were silanized to enhance the chemical interaction and adhesive bonding strength to PCL. The surface characteristics were microstructurally analyzed by confocal microscopy, SEM and sessile drop method with regard to their surface texturing. The adhesive bonding strength to PCL was determined by single-lap compressive shear tests to find an optimum mechanical surface texturing.

## 2. Materials and Methods

### 2.1. Fabrication of Surface-Textured HAp Ceramics by Micro-Transfer Molding

**Figure 1:**
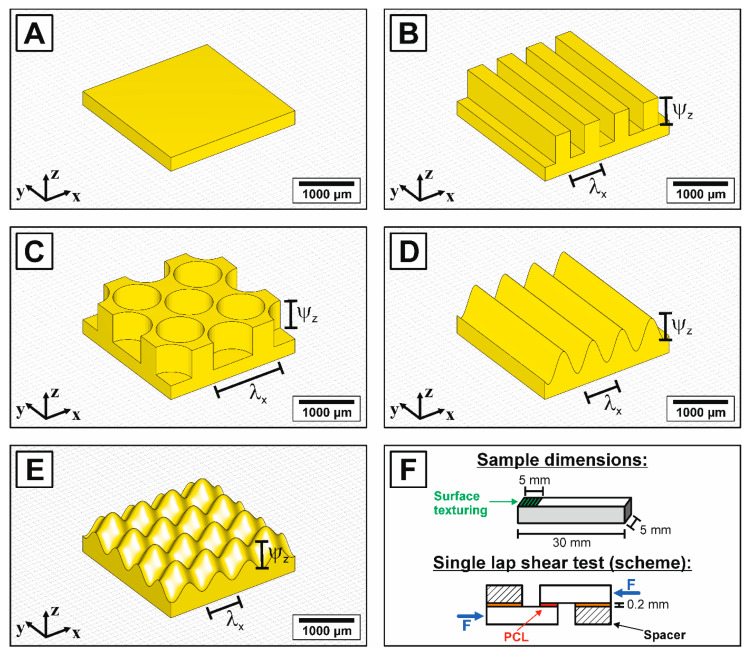
Schematic representation of the realized surface textures on a cross-sectional area of 2.35 mm × 2.35 mm: non-textured reference (**A**), linear grooves (**B**), cylindric pits (**C**), linear waves (**D**) and Gaussian hills (**E**). Real sample dimensions of the fabricated test bars (30 mm × 5 mm × 5 mm) with a surface texture of 5 mm × 5 mm used for the single-lap shear testing and schematic testing setup of the applied single-lap shear test to evaluate the adhesive bonding strength of the surface-textured HAp ceramics to polycaprolactone (PCL) (**F**).

Surface-textured HAp ceramics with sub-millimeter periodic macro-texturing were fabricated by a micro transfer-molding technique, whose process steps are described in detail in [37,38,39]. Four different types of surface textures with an identical structuring depth of 500 µm (equal to an amplitude of ψ_z_/2 = 250 µm) and identical structuring spacing (or wavelength) of λ_x_ = 550 µm were generated using open source software blender (v. 2.82a, Stichting Blender Foundation, Amsterdam, The Netherlands). The generated CAD files were afterwards 3D-printed using an stereolithographic 3D-printer (printer: Digitalwax^®^ 028J, resin: Fusia DC700, both: DWS S.r.l., Zanè, Italy) with a z-layer resolution of 20 µm. Figure 1 shows representative CAD files of the non-textured reference (A) and the four realized surface-textured (B–E), the corresponding structural parameters are described in Table 1. The 3D-printed forms were molded with a polydimethylsiloxane (PDMS) elastomer (Elastosil M 4643 A/B, Wacker Chemie AG, München, Germany) to obtain the negative silicon casting molds for the transfer-molding process. The preparation of the utilized HAp raw powder (04238, Sigma-Aldrich Corp., St. Louis, MO, USA), the powder hydrophobization and the processing to the HAp transfer mold feedstock was identical to previous published work, to which we refer for a detailed description of the preparation process [37,38]. The utilized HAp feedstock contained a solid loading of 50.0 Vol% hydrophobized HAp powder, 44.9 Vol% paraffin wax (Granopent P, Carl Roth GmbH, Karlsruhe, Germany) and 5.1 Vol% carnauba wax (naturfarben, Carl Roth GmbH, Karlsruhe, Germany). The transfer molding was performed at 120 °C supported by applying a gentle vacuum (<10 Pa) to degas the dispersion and to ensure a contour and shape accurate molding of the sub-millimeter surface texturing. The samples were wick debinded on a porous mullite substrate (Annamullit^®^88, Compagnie de Saint-Gobain S.A., Courbevoie, France) and afterwards sintered at 1250 °C for 2 h.

### 2.2. Surface Treatments of the Sintered (Textured) HAp Ceramics

An additional surface texturing on a micron and nanometer scale was achieved by a subsequent acid etching and silanization process. Non-textured reference samples were polished (P) finishing with a 1 µm diamond suspension and afterwards etched using HCl (H) and tartaric acid (T). For the HCl-etching, one HAp sample (30 mm × 5 mm × 5 mm) was immersed in 10 mL of 32% hydrochloric acid (HCl, Supelco^®^, Merck KGaA, Darmstadt, Germany) for 2 min at room temperature (25 °C). For the etching with tartaric acid (L(+)-tartaric acid, CAS Number: 87-69-4, Merck KGaA, Darmstadt, Germany), one HAp sample (identical dimensions) was immersed in 10 mL of 0.1 molar tartaric acid solution for 5 h at a constant temperature of 5 °C. After the etching processes, all samples were thoroughly rinsed and cleaned with double distilled water to completely remove the acid residuals. The tartaric acid-etched samples were afterwards dried at 180 °C for 15 min to drain out the crystal water of the as-precipitated Ca-tartrate crystals [40]. Additionally, samples were silanized according to [41] in a 1.5 Vol% 3-aminopropyltriethoxysilane (APTES, 98% purity, abcr GmbH, Karlsruhe, Germany) solution using anhydrous toluene (99.5% purity, VWR International LLC, Radnor, PA, USA) as a solvent. Before use, the toluene was dehydrated for 24 h by adding 100 g/L of a 4 Å molecular sieve (Supelco^®^, Merck KGaA, Darmstadt, Germany). The silanization reaction was performed in a round-bottomed flask at 120 °C to chemically bond the APTES on the HAp ceramic surface by a dissociation reaction [41], which was indirectly heated by a silicon oil bath. After a reaction time of 5 h, the silanized samples were thoroughly cleaned with pure toluene and afterwards rinsed with pure ethanol (≥99.8% purity, VWR International LLC, Radnor, PA, USA). A hierarchical combination of the mentioned surface textures was achieved by applying the surface treatments (etching with hydrochloric acid and silanization) to the macro-surface-textured HAp ceramics of texture E, Table 1.

### 2.3. Characterization

The surface topography including the contour and shape accuracy as well as the surface roughness of the surface-textured HAp ceramics were analyzed with a laser scanning microscope (VK-X160, Keyence Corp., Osaka, Japan) equipped with a red laser (λ = 658 nm), a theoretical z-resolution of 5 nm and xy-resolution of 10 nm. The surface roughness was calculated from multiple profile measurements analyzing a measuring distance of 3 mm for each sample type. The cut-off wavelengths (λ_c_, λ_s_) were chosen depending on the measured surface roughness according to DIN EN ISO 4288:1998-04 and DIN EN ISO 4287:2010-07 [42,43]. Additionally, the surface coefficient was determined as the ratio of the real-surface area to the scanned cross-sectional area and was then compared to the theoretical surface coefficient, which was calculated from the CAD models. The dimensional deviations between the sintered surfaces (derived from the laser scanning microscopy) and the CAD models (considering the anisotropic sintering shrinkage of the individual texture types) were determined with the open-source visual inspection software GOM Inspect (GOM GmbH, Braunschweig, Germany) using best-fit alignment.

To determine the total surface energy including the polar and disperse fractions, the contact angle was determined by sessile drop method according to DIN EN 828:2013-4 and DIN 55660-2:2011-12 using distilled water (H_2_O), glycerol (C_3_H_8_O_3_) and diiodomethane (CH_2_I_2_) as partially wetting measuring liquids [44,45]. The surface energies and its fractions were calculated from the contact angles using Lifshitz–van der Waals acid–base (LWAB) theory [46,47]. The total surface energy ($\gamma_{s}^{tot}$) is given by the nonpolar electrodynamic Lifshitz–van der Waals ($\gamma_{s}^{LW}$) and the polar Lewis acid–base interactions ($\gamma_{s}^{AB}$) with its acidic ($\gamma_{s}^{+}$) and basic ($\gamma_{s}^{-}$) component (Equation (1)) [46,47].

(1) $$\gamma_{s}^{tot}=\gamma_{s}^{LW}+\gamma_{s}^{AB}=\gamma_{s}^{LW}+2\cdot \sqrt{\gamma_{s}^{+}\cdot \gamma_{s}^{-}}$$

The three unknown variables of Equation (1) ($\gamma_{s}^{LW}$,$\;\gamma_{s}^{+}$,$\;\gamma_{s}^{-}$) were determined by solving the linear system of equations defined by the three measuring liquids and corresponding contact angles using the LWAB-modified Young–Dupré equation (Equation (2)) inserting the known surface tension components of the liquid ($\gamma_{l}^{LW},\gamma_{l}^{+},\;\gamma_{l}^{-}$) given in Table 2 [46,47]:

(2) $$W_{A}=\gamma_{l}^{tot}(1+\cos\theta )=2\cdot \left(\sqrt{\gamma_{s}^{LW}\cdot \gamma_{l}^{LW}}+\sqrt{\gamma_{s}^{-}\cdot \gamma_{l}^{+}}+\sqrt{\gamma_{s}^{+}\cdot \gamma_{l}^{-}}\right)$$

From Equation (2), the work of adhesion (*W_A_*) can be additionally derived inserting the measured contact angle (θ) between the solid (*s*) and liquid (*l*).

The adhesive bonding strengths between the surface-textured HAp ceramics and the bioresorbable polymer poly-ε-caprolactone (PCL) were determined in single-lap, thick-adherend compressive shear tests using a universal testing machine (Instron 5565, Instron GmbH, Pfungstadt, Germany) according to DIN EN 1465:2009-07 and ISO 11003-2:2019-06 [49,50]. For the testing, the specimen and adhesive surface dimensions of [49,50] were adapted to a ceramic suitable construction. Test bars with dimensions of 5 mm × 5 mm × 30 mm and an integrated texturing with a size of 5 mm × 5 mm × 0.5 mm at one end of the top surface were fabricated (see illustration of Figure 1F). Well-defined PCL (Facilan^TM^Ortho, 3D4MAKERS, Haarlem, The Netherlands) platelets (5 mm × 5 mm × 0.5–0.9 mm) were 3D-printed with a fused deposition modeling (FDM)FDM printer (Ultimakers 2+, Ultimaker B.V., Ultrecht, The Netherlands) using a nozzle diameter of 0.4 mm, printing speed of 20 mm·s^−1^ and z-layer resolution of 0.2 mm. The printing process was utilized to adapt the thickness of the PCL platelets individually to the displaced volume of the different surface texture types. Two HAp samples were bonded together at their textured surface with one molten PCL platelet by uniaxial warm pressing. A temperature of 180 °C was applied for 15 min with a decent external uniaxial pressure of 0.003 MPa, resulting in a full polymer coverage for all texturing types and a constant PCL thickness of 200 µm. Ten samples of each surface-textured type were tested using a crosshead speed of 10 mm·min^−1^.

### 2.4. Statistical Analysis

The dimensional accuracy of each macro-surface texturing was determined as the deviation from the CAD model (with the visual inspection software GOM) determining the mean and 5σ-standard deviation (equal to 99.977% confidence interval). The individual compressive shear strengths were plotted as mean values with corresponding standard deviation. A non-parametric, two-tailed Wilcoxon–Mann–Whitney test (*U* test) was applied to distinguish the sample series. *p*-values of at least 0.05 (or 0.01) were considered as significant and denoted as *p* < 0.05 or *p* < 0.01, respectively.

## 3. Results and Discussion

### 3.1. Microstructural Characterization of Macro-Surface-Textured HAp Ceramics

**Figure 2:**
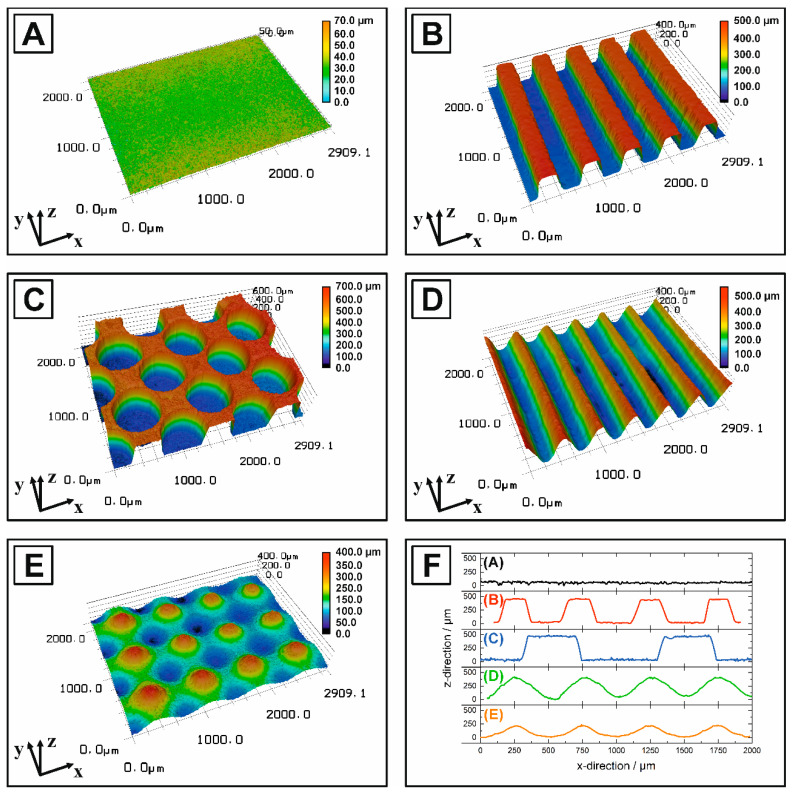
Surface topography of the sintered HAp with different macro-surface texturings analyzed by confocal microscopy: non-textured reference (**A**), linear grooves (**B**), cylindric pits (**C**), linear waves (**D**), Gaussian hills (**E**) and the corresponding representative height profiles showing the wavelength and amplitude of each surface texturing (**F**).

The tailored manipulation of surfaces represents one of the current aims to improve the performance and durability of implants [13,15,16,17,18,19,20,21]. Based on the high hardness, chemical stability and brittleness of ceramics, a near net shape manufacturing allowing a controlled surface texturing from the design stage is preferable to a subsequent hard machining process [1]. Moreover, the complexity of surface morphologies realized by hard machining is limited. In this work, we therefore present the feasibility utilizing a stereolithography-based micro-transfer molding technique to generate HAp ceramics with customized, periodic surface textures in a submillimeter range. The surface topographies of the sintered ceramics with four different macro-surface textures are shown in Figure 2. A successful positive replica with a contour-accurate molding of the generated CAD models (Figure 1) could be realized for all surface morphologies including the geometries of cylindrical pits, linear grooves and waves, as well as Gaussian hills. The corresponding height profiles (Figure 2F) and the R_sm_-value of Table 3 show an identical wavelength along the x-direction for all structures, excluding the double wavelength of texture type C. However, a loss in the z-resolution causing a decreased texturing depth (ψ_z_) indicated by the R_c_-values of Table 3 was observed for the texture types with non-vertical walls (surface texture D + E).

**Figure 3:**
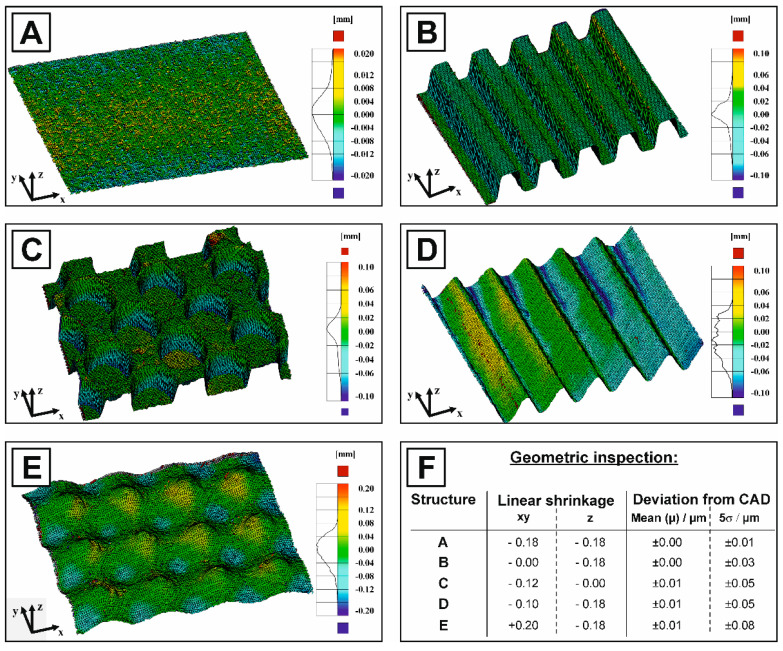
Nominal–actual surface comparison of each texturing (**A**–**E**), showing the dimensional deviations between the real sintered HAp surfaces with macroscopic surface texturing and CAD models (considering the anisotropic shrinkage) by colored heat maps and corresponding histogram. The color legend has the following meaning: blue = negative deviation, real surface is below CAD surface; green = no deviation; red = positive deviation, real surface is above the CAD surface. (**F**) shows the assumptions made regarding anisotropic shrinkage and the determined dimensional deviations (mean- and 5σ-deviation) for each texturing type.

Although many texturing techniques have been established to generate well-defined periodic surface patterns, the accuracies of the generated surfaces are rarely investigated [1,13,15,16,17]. Visual inspection software offer the possibility to quantify the dimensional deviations and accuracy to the designed CAD model, but has never been used for surface-textured ceramics [51,52]. For that reason, the visual inspection software GOM was used to quantify the accuracy of the utilized micro-transfer molding technique by determining the dimensional deviations between the sintered surface textures and the corresponding CAD models considering the real sintering shrinkage. Although a nearly isotropic linear shrinkage of 18 % was measured for the sample bars on the macroscopic scale, the anisotropic patterns of the different surface textures led to an anisotropic shrinkage in the individual spatial directions on a micron scale. The real individual shrinkages were obtained from the laser scanning microscopy images (height profiles, Figure 2F) and then implemented to adapt the CAD models to the real sintered surfaces. Figure 3 shows the corresponding nominal-actual surface comparison highlighting the dimensional deviations by a colored heat map (blue = negative deviation, real surface is below CAD surface; green = no deviation, red = positive deviation, real surface is above the CAD surface) and an associated histogram. A high accuracy molding was achieved on the entire surface (5 × 5 mm^2^) for all surface texture types with a 5σ deviation of ≤±80 µm, which is close to the theoretical resolution of the used 3D printer (∆_xy_ = 22 µm for the laser spot and ∆_z_ = 20 µm for the layer height). The dimensional deviations of texture D and E can primarily be attributed to a loss of print resolution for curvature surfaces. Curvatures and rounded edges are stepwisely divided into layers during the slicing process, causing a geometric deviation, which in this case led to excessive curing of surrounding monomer solution. Additionally, residual uncured monomer, which could not be completely removed from the small cavities during the washing process and is afterwards cured in the subsequent UV curing. The inaccuracies of the SLA process (overexposure, residual monomer, sagging/warping and peeling effects [51]) are most crucial as they are transferred to the molded silicon form and subsequently to the molded ceramic part.

The generation of surface textures is not only associated with a modification of the surface morphology but also with a change of the surface area. The HAp surface represents the contact area for potential biological events (protein adsorption, cell adhesion and bone ongrowth) [2,4,5,7,8,9,10] and additionally determines the component adhesion in composites (here HAp/PCL) [17,18,19,20,21]. Besides the four realized surface morphologies, the associated contact surface area is one of the most important surface characteristics affecting the adhesive bonding strength. The adhesive bonding strength is strongly dependent on the adhesive surface and increases with increasing overlap area of the bond as long as the fracture occurs in the adhesive joint and not in the joined parts [53]. Thus, the determination of the real contact surface area of each macro-texturing is mandatory to evaluate its effect on the adhesive bonding strength. The normalized surface area (surface coefficient) was experimentally derived from the laser scanning microscopy and is comparatively shown in Table 3 to the theoretical surface coefficient determined from the CAD models. The surface coefficient (*S_A_*) is a surface multiplication factor representing the ratio of the real surface area to the examined cross-sectional area. For an idealized flat surface, the surface area equals the cross-sectional area (*S_A_ = 1*); a *S_A_ > 1* represents a surface enlargement. All four macroscopic surface texturings (B–E) exhibited significantly higher surface than the non-textured (A) and polished reference (P), which exhibited a $S_{A}^{exp.}=\;$1.1–1.3. A maximum *S_A_* was obtained for texture types with vertical walls with $S_{A}^{exp.}=3.5$ for the cylindric pits and $S_{A}^{exp.}=3.3$ for the linear grooves. This demonstratively corresponds to an increase in the surface by a factor of 3.5 on the identical cross-sectional area. Thus, the macroscopic surface texturing offers great potential to increase the real contact surface area between HAp and PCL in order to maximize the adhesive bonding strength. Although the theoretical and experimental *S_A_* were generally in a very good agreement for all texturing types, the experimental surface coefficient was always higher than the theoretical one ($S_{A}^{exp.}>\;S_{A}^{theo.}$). This can be attributed to a certain roughness on the sintered ceramic surface, which was also shown for the non-textured reference A with a $S_{A}^{exp.}\;$= 1.3 instead of the theoretical value of $S_{A}^{theo.}\;$= 1.0.

### 3.2. Microstructural Characterization of the Micro- and Nano-Surface-Textured HAp Ceramics

**Figure 4:**
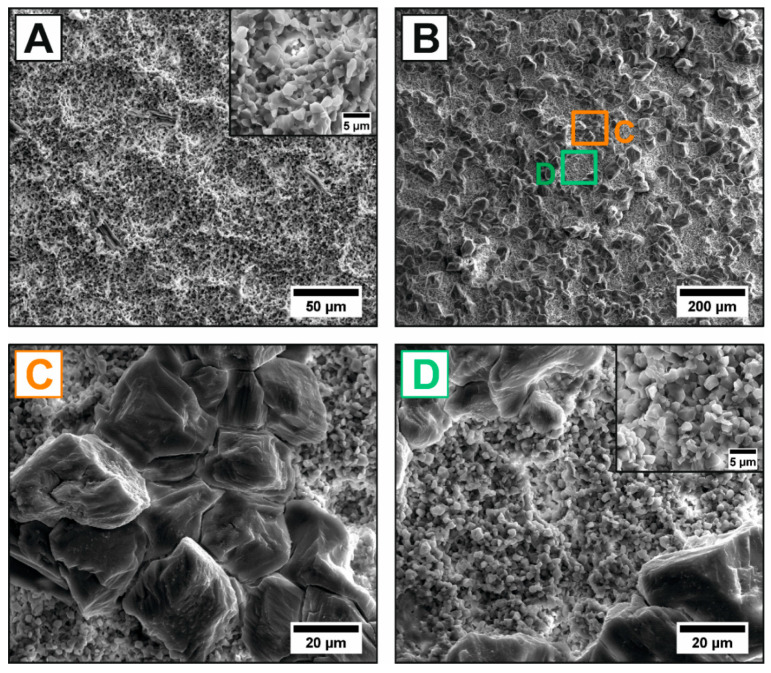
Microstructure of the acid-etched HAp surfaces showing a surface roughening for the subtractive etching-process of HCl (**A**). A significantly higher surface roughness was observed for the tartaric acid-etched samples (**B**–**D**), combining an additive precipitation of Ca-tartrate crystals on the HAp surface (**C**) and subtractive etching of the HAp matrix (**D**). The two etching treatments showed no influence on the initial grain size (high-resolution image section of (**A**,**D**)).

In order to optimize the adhesive bonding strength to PCL, a hierarchical surface texturing of HAp was introduced by a subsequent acid etching using hydrochloric acid (H) and, for the first time, tartaric acid (T). Etching is a well-established technique in the dental industry to roughen the surface on a micron scale by dissolving the material and simultaneously functionalize the surface by generating activated functional groups (e.g., hydroxylation = creation of OH^−^ groups) [9,17,18,19,20,54]. In contrast to the chemically highly stable alumina [54] and zirconia [20] ceramics, the etching of the calcium-phosphate-based HAp can be already carried out in slightly acidic environments as the dissolution starts occurring at pH < 7.2 [55]. Not only the mechanisms of the HAp dissolution itself remain controversial [55], also the ongoing etching process was shown to depend on the utilized acid type [56]. The etching of HAp with HCl (H, Figure 4A) and tartaric acid (T, Figure 4B–D) resulted in different microstructures and surface topographies, shown in the SEM micrographs of Figure 4. For the etching with HCl, a pure dissolution of the HAp ceramic, characterized by the typical etch-pits formation, was observed. The net reaction for the subtractive etching of HAp with strong acids (*HA* = HCl, HNO_3_, HBr, …) with an easily soluble anion (*A^−^* = Cl^−^, NO_3_^−^, Br^−^, …) can be described by Equation (3), assuming a complete HAp dissolution:

(3) $$\begin{array}{cc} Ca_{5}{\left(PO_{4}\right)}_{3}{\left(OH\right)}_{\left(s\right)} & +H_{3}O^{+}{}_{\left(aq\right)}+A^{-}{}_{\left(aq\right)}\\ & \to 5\;Ca^{2+}{}_{\left(aq\right)}+3\;PO_{4}{{}^{3-}}_{\left(aq\right)}+2\;H_{2}O_{\left(l\right)}+A^{-}{}_{\left(aq\right)} \end{array}$$

In addition to the pure HAp dissolution, the etching with tartaric acid resulted in the nucleation and growth of adherent Ca-tartrate crystals (CaC_4_H_4_O_6_, size ≈ 50 µm) on the etched HAp surface. The Ca-tartrate crystals were homogenously but isolate distributed on the entire surface without forming a continuous coating layer, which agrees well with the observations carried out on marble (CaCO_3_) substrates [57]. Some carboxylic acids (R-COOH) with two or more carboxylic groups such as tartaric acid were shown to form water-insoluble crystalline complexes by chemical bonding between the deprotonated carboxylates (R-COO^−^) and released/free Ca^2+^-ions [56,58,59]. A single Ca^2+^-ion is capable to bond two carboxylic groups from two acid molecules to form 3D-crystalline structures. The general corresponding net reaction for carboxylic acids with HAp can be described by Equation (4):

(4) $$\begin{array}{cc} Ca_{5}{\left(PO_{4}\right)}_{3}{\left(OH\right)}_{\left(s\right)} & +10\;H_{3}O^{+}{}_{\left(aq\right)}+10\;R-COO^{-}{}_{\left(aq\right)}\;\\ & \to 5\;{\left[Ca^{2+}\cdots {\left(R-COO^{-}\right)}_{2}\right]}_{\left(s\right)}+3\;H_{3}PO_{4}{}_{\left(aq\right)}+11\;H_{2}O_{\left(l\right)} \end{array}$$

**Figure 5:**
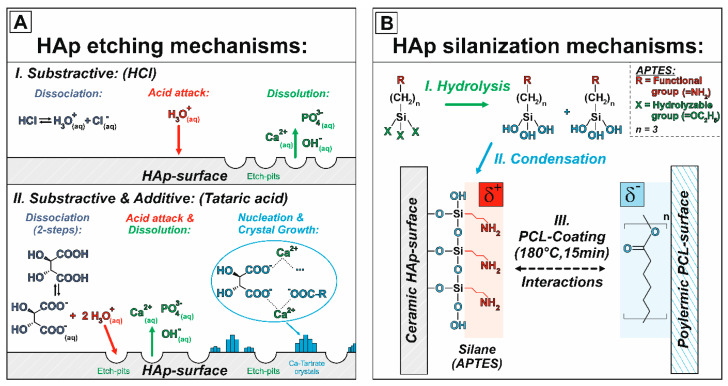
Schematic reaction mechanisms of the acid etchings (**A**) and the silane coupling (**B**) with the ceramic HAp surface. For HCl, the etching of HAp can be described by a simple dissolution process (subtractive etching). In the case of carboxylic acids with two or more carboxylic-groups (here tartaric acid), the chemical reaction between the deprotonated carboxylates and released Ca^2+^-ions results in an additional nucleation and crystal growth of adherent, water-insoluble Ca-carboxylate complexes (here Ca-tartrate crystals) on the HAp surface. The etching of HAp with carboxylic acids is therefore a mixture of subtractive dissolution and additive precipitation of crystals. The mechanism of the silane coupling (here 3-aminopropyltriethoxysilane (APTES)) can be described by a condensation reaction, forming covalent bonds between the silane and the functional groups of the HAp surface. The silane’s functional groups “R” (here the amino group of APTES) can then improve the adhesive bonding strength between HAp and polymeric PCL by forming dipole–dipole interactions or chemical bonds.

The chemical reaction of tartaric acid with HAp is therefore a mixture of a subtractive dissolution of HAp by the acid protons (H^+^) and an additive precipitation of Ca-tartrate crystals, which were also proven in the SEM micrographs of Figure 4C,D. The two reaction mechanisms of the subtractive etching and additive precipitation are schematically visualized in Figure 5A. The ability of carboxylic acids to form thermodynamically stable crystals on HAp surfaces depends on the solubility of the corresponding Ca-salt [56]. The as-formed Ca-tartrate crystals can be converted to CaCO_3_ (T = 667 °C) or CaO (T = 807 °C) by a subsequent heat treatment [40] offering great potential to further tune the biological response in-vitro and –vivo. Continuous ${\left[Ca^{2+}\cdots {\left(R-COO^{-}\right)}_{2}\right]}_{x_{\left(s\right)}}$ coatings can be obtained by using oxalic acid [57]. However, in order to maximize the surface roughness for a better PCL adhesion, in this work the discontinuous Ca-tartrate crystals formed from the tartaric acid were preferred. Both acid etchings were associated with a significant surface roughening from an initial R_a_^F^ = 0.98 µm for the as-fired (F) surface to R_a_^H^ = 2.93 µm for HCl (H) and R_a_^T^ = 9.80 µm for tartaric acid (T). This shows that the additional precipitation (nucleation and growth of Ca-salt crystals) is more effective to create rough surfaces than a subtractive etching alone. The crystallite size can be controlled by changing the etching conditions (pH, concentration, temperature or time). The roughness achieved by etching is highly interesting for an improved adhesion of cells, which were proven for a similar surface roughness generated by grinding (R_a_ ~ 2.8–4.7 µm) [7]. In order to preserve the well-defined surface of the periodic macro-texturings, in this work the etching with HCl was preferred for the hierarchical surface texturing.

On the atomic scale, both acid etchings additionally introduced a significant change of the HAp surface chemistry, shown in the change of the wettability and thus the surface energies. During the HAp dissolution free hydroxy groups (OH^−^-groups) are generated at the surface, which either can function as reactive sites for the further silanization or form chemical bonds with the polymeric PCL by esterification. The subsequent silanization with ATPES generated positively charged amino groups (NH_2_-groups) at the surface [36]. The reaction mechanism of APTES with the ceramic HAp surface is visualized in Figure 5B and is based on the chemical bonding to (as-generated) OH^−^-surface sites. The positively charged NH_2_-groups of APTES improve the adhesive bonding to PCL mainly by dipole-dipole-interactions and by potentially forming covalent amid-bondings (R-CO-NH-R’) with carboxylic end-groups or ruptured ester-groups of the PCL chains, thermally induced by the warm pressing at 180 °C, 15 min [60,61,62]. The modification of the surface chemistry was investigated by determining the total surface energy ($\gamma_{s}^{tot})$ and its non-polar ($\gamma_{s}^{LW}$) and polar ($\gamma_{s}^{AB},\;\gamma_{s}^{+},\gamma_{s}^{-}$) fractions solving the linear equation system of the LWAB-modified Young–Dupré equation (Equation (2)) for three different partially wetting liquids (Table 2). The calculated surface energies are shown in Table 4. In comparison to the ceramic HAp surface, the surface energy of the polymeric PCL is predominantly influenced by its non-polar component ($\gamma_{s}^{LW}$) and thus give PCL a hydrophobic surface. However, the inherent functional ester groups provide PCL a comparatively high polar fraction for polymers. An acceptable agreement with reported surface energy values for pure HAp and PCL was obtained [63,64]. In the case of HAp, the deviations, especially of the dispersive component ($\gamma_{s}^{LW}\left(HAp\right)$), can be attributed to the usage of different measuring liquids [65] and differing sample conditions (in [64] powder were used instead of sintered ceramics). For the polymeric PCL the deviations mainly rely on the degree of polymerization, which additionally is influenced by the processing parameters (thermal treatments, here: extrusion during the 3D printing and subsequent warm-pressing process).

The surface functionalization by the acid etching and silane treatment influenced the wetting and thus the surface properties of HAp. On the one hand both acid etchings (H, T) significantly increased the polar surface energy component ($\gamma_{s}^{AB}$) and thus make the HAp surface more hydrophilic, which was more effective for the combined subtractive and additive etching of tartaric acid. On the other hand, the silanization with APTES resulted in an increased disperse fraction ($\gamma_{s}^{LW}$) creating a hydrophobic surface, which can be tailored by changing the utilized silane type [36,41]. An effective strategy to improve the physical adhesion (*W_A_*) to a polymer is to maximize the total surface energy ($\gamma_{s}^{tot}$) of the ceramic, calculated by Equation (1). This was achieved for the acid etchings, for which $\gamma_{s}^{tot}$ was increased by a factor of 1.6 for HCl and 1.84 for tartaric acid. The hydrophobic silanization caused a decrease in $\gamma_{s}^{tot}$ compared to HAp (factor 0.84). The physical adhesion energy (*W_A_*) to molten PCL was calculated using the LWAB theory inserting the values of Table 4 into Equation (2). Corresponding to $\gamma_{s}^{tot}$, the physical adhesion could be improved by the acid etching and showed a maximum for tartaric acid etched HAp with 104.7 mJ/m^2^. Although the physical adhesion decreased after the APTES silanization, the discussed dipole–dipole interactions and potential formation of chemical bonds to PCL will improve the experimental adhesive bonding strength.

### 3.3. Adhesive Bonding Strength of Hierarchical Surface-Textured HAp to PCL

**Figure 6:**
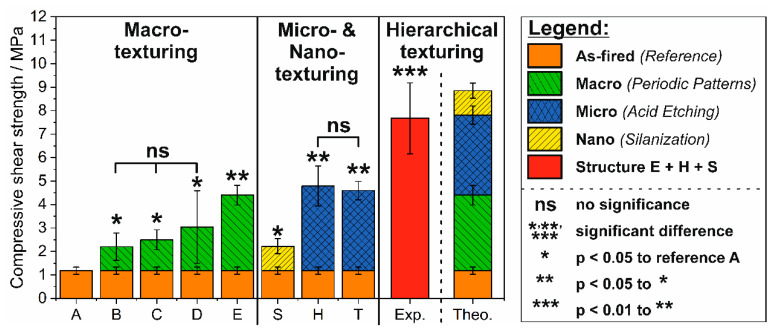
Adhesive bonding strength between hierarchical surface-textured HAp and PCL. Left side shows bonding strength of the well-defined macro-texturings B–E (green color) and non-textured reference (A). The silanized nano-texturing (yellow color) and the HCl (H) and tartaric acid (T) etched micro-texturings (blue color) are shown in the middle. The hierarchical texturing combining the macro-texturing E with a subsequent HCl etching and silanization (red column) are shown on the right side. The rightest column shows a theoretical model assuming a non-weighted additive composition of the individual contributions of the macro texture type E, HCl-etched (H) and silanized (S).

The poor interface strength in HAp/PCL composites is a remaining obstacle for their clinical use as implants [26,28,31]. The interface strength of composites is influenced by their component adhesion, which may have a mechanical, physical, chemical or combined origin. In this work, a novel hierarchical HAp surface texturing was developed to overcome this issue by making use of all mentioned mechanisms. The adhesive bonding strength between non-textured HAp and PCL was so far investigated by 4-point bending [28] and T-peel test [26,66]. In this work, the adhesive bonding strength was determined by compressive shear tests in dependence of the applied macro-, micro- and nano-texturing, shown in Figure 6. To highlight the respective improvements of each texturing type, the fraction of the initial interfacial strength of the non-textured reference (texture type A) was colored separately.

**Figure 7:**
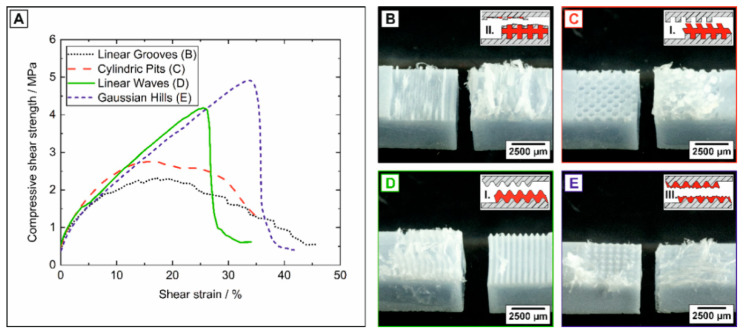
Representative stress–strain curves (**A**), corresponding fracture surfaces and failure types (I.–III.) from the compressive shear tests between the macro-surface-textured HAp and PCL (**B**–**E**).

With a value of 1.2 ± 0.2 MPa the initial HAp/PCL interfacial strength (non-textured reference A) is comparatively lower than the reported value of 9 ± 2 MPa for 4-point bending [28]. The differences of the bonding strengths can be attributed to the individual stress conditions generated by the two testing-setups and specimen dimensions (bonding layer and adherend thickness) [26,28,66,67,68]. Whereas a mixture of tensile and compressive stresses occur in the adhesive layer during 4-point bending; inhomogeneously distributed but pure shear stresses are generated by the single-lap (compressive) shear tests utilized in this work [67,68]. For high performance adhesives, the failure stress ratio is given by 70:31:31:1 (MPa) for compression:tension:shear:peel stresses [68], showing that for pure shear stresses failure will occur at lower stress conditions than for bending moment generated tensile and compressive stresses. A significant improvement of the adhesive bonding strength was observed for all texturing types from macro- to nano-texturing independent of applied dimensional scale, Figure 6. For the periodic macro-texturings, the origin of the interfacial strength enhancement is the improved mechanical adhesion provided by an enlarged contact surface and a mechanical interlocking ability [69,70]. Interestingly, the highest bonding strength improvements by a factor of 2.6 and 3.7 (compared to non-textured A) were not achieved for the textures with the highest surface coefficients and sharp edges (B + C), but instead for texture type D and E, respectively (referring to the *S_A_-values* of Table 2). This shows that the surface enlargement is not the dominant factor for the mechanical adhesion, but moreover the surface morphologies determine the fracture mechanism, failure behavior and thus the adhesive bonding strength. Representative stress–strain curves and the corresponding fracture surfaces with their schematic failure mechanisms are shown in Figure 7. Three different failure mechanisms (I–III, Figure 7) were observed for the macro-texturings. A pure or substrate-near adhesion failure (failure type I) at the HAp/PCL interface frequently occurred for the non-textured reference (A, not shown here), cylindric pits (C) and linear waves (D). The adhesion failure is characterized by a one-sided peeling of the PCL layer from the textured HAp ceramics caused by the poor initial interface strength between HAp and PCL [53]. Contrary to the non-textured reference, the macro-texturings provide a mechanical interlocking between the textured HAp ceramic and PCL layer [69,70]. Thus, the peeling is associated with a segmental polymeric pull-out from the texturing and a massive plastic deformation in the PCL layer (Figure 7C,D) resulting in an enhanced interfacial strength. In contrast, the macro-texturings of the linear grooves (B) frequently showed a substrate-near cohesion fracture (failure type II), for which the fracture propagated trough the PCL and locally through the thin, sharp-edged ceramic walls of the texturing, Figure 7B. Failure type II can be attributed to the non-suitable ceramic design of sharp edges, as for the identical texturing with rounded edges (linear waves D) the failure did not occur in the ceramic. The Gaussian hill (E) texturings fractured by a pure cohesive failure (failure type III.) in the PCL layer, showing that the HAp/PCL interface was not the weakest link for this macro-texturing. The transition from a pure adhesion failure at the interface without any interlocking effects to a pure cohesion failure in the PCL by introducing periodic macro textures, proves that the HAp/PCL interface strength could be effectively improved just by enhancing the mechanical interlocking and adhesion.

The acid-etched micro-texturings showed even higher interfacial strengths with improvement factors of 3.9 and 4.1 for tartaric acid (T) and HCl (H), respectively. The discussed surface roughening and surface functionalization of the acid treatments were shown to effectively improve both the mechanical and mainly the physical adhesion. The comparatively smallest improvement was achieved with the silanized samples (S), for which the initial strength could still be almost doubled (factor of 1.9) by forming the discussed dipole–dipole interactions and chemical bonds between the silanized HAp and PCL. The different mechanisms of action and orders of magnitude of the applied surface texturing allow the free combination of each technique to achieve a hierarchical surface texturing with negligible negative interactions. For that purpose, the best of all individual texturings were chosen to generate a hierarchical surface-textured HAp ceramic, combining the mechanical interlocking of texture type E with the improved mechanical and physical adhesion of a subsequent HCl etching and the enhanced chemical adhesion of the silanization (E + H + S). For the hierarchical surface-textured HAp a maximum adhesive bonding strength of 7.7 ± 1.5 MPa could be achieved, which is 6.5 times higher than the initial interfacial strength of the non-textured reference (A). To estimate the interfacial bonding strength of hierarchical textured surfaces, a simple theoretical model was derived, assuming an additive composition of the individual contributions with no interactions, Figure 6. The total adhesive strength can then be described by the sum of the individual adhesion components (*W_i_*) in Equation (5):

(5) $$W_{tot.}=\overset{n}{\underset{i=1}{\sum }}W_{i}=\;W_{M}+\;W_{A}+W_{C}$$

By inserting the applied mechanisms of this work, including the mechanical (*W_M_*), physical (*W_A_*) and chemical adhesion (*W_C_*) component, the experimental strength was slightly lower than the theoretical model of Equation (5), but still showed an acceptable agreement with regard to the large standard deviations. For a more accurate description, the interactions between the mutual individual techniques have to be considered. The subsequent silanization for instances influence the physical adhesion of the acid etching as it was shown in Table 3 for T + S. Regardless of that fact, Equation (5) provides a first valid estimation of the adhesive bonding strength for hierarchical textured surfaces. Based on these results, we have shown that the developed hierarchical surface texturing approach can be used to tailor the surface area and morphology in order to improve the interface strength in bonding applications.

## 4. Conclusions

Hydroxyapatite ceramics with a hierarchical surface texturing were fabricated in order to improve the adhesive bonding strength in ceramic–polymer composites (HAp/PCL) used as potential implants. Four types of well-defined, periodic macro-texturings were realized on a submillimeter scale by a stereolithography based ceramic micro-transfer molding technique. The technique allows a morphology independent near-net shaping with accuracies < 80 µm. The generated macro-texturings (linear grooves, cylindric pits, linear waves, Gaussian hills) provided a mechanical interlocking ability and thus enhanced the mechanical adhesion between HAp and PCL. An additional surface roughening and functionalization on a micron scale was obtained by a subsequent etching with hydrochloric and tartaric acid (micro-texturing). While for HCl the etching could be described by a simple dissolution process, the etching mechanism of tartaric acid was a mixture of HAp dissolution and precipitation of Ca-tartrate crystals on the HAp surface. Both acid etchings significantly increased the surface roughness and surface energy (especially the polar fraction) and thus provided an improved mechanical as well as physical adhesion. A nano-texturing was realized by an ensuing silanization generating positively charged amino groups on the HAp surface. The silane coupling enabled a chemical interaction between the normally chemical inert HAp surface and the polymeric PCL by forming dipole–dipole interactions and covalent bonds and thus improves the chemical adhesion. Based on the different effect mechanisms and order of magnitude, the mentioned surface texturing techniques can be deliberately combined. The hierarchical surface-textured HAp showed with 7.7 ± 1.5 MPa a 6.5 higher adhesive bonding strength compared to the non-textured HAp reference. Thus, hierarchical surface texturing is an efficient way to improve the performance of HAp/PCL composites and thus the lifetime of potential implants.

## Figures and Tables

**Table 1:** Types, corresponding structural characteristics and realized surface treatments of the fabricated hydroxyapatite (HAp) surface texturings. All dimensional specifications refer to the computer-aided designed (CAD) models.

Texturing Type	Surface Morphology	Structuring Spacing (Wavelength λ)		Structuring Depth (2 × Amplitude ψ)	Tested Surface Treatments
		**λ_x_**	**λ_y_**	**Ψ_z_**	**
		**/µm**	**/µm**	**/µm**	
A	Non-textured	-	-	500	F, P, H, T, S, T + S
B	Linear grooves	550	-	500	F
C	Cylindric pits	1100	780	500	F
D	Linear waves *	550	-	500	F
E	Gaussian hills *	550	390	500	F, H+S

* Mathematical description of texture D and E is given by z_D_ = ψ·sin(λ·x) and z_E_ = ψ·[sin(λ/2·(x + y))·cos(λ/2·(x − y))] with ψ_z_ = 0.25 and λ = 2π/λ_x_ = 11.5. ** Tested surface treatments: F: as-fired, P: polished with 1 µm diamond suspension, S: silanized, H: HCl-etched, T: tartaric acid-etched, T + S: etched with tartaric acid and afterwards silanized.

**Table 2:** Surface energies of the measuring liquids used for Lifshitz–van der Waals acid–base (LWAB) theory according to [48].

Measuring Liquids	Reference	Surface Energies (γ)			
		γ^tot^	γ^LW^	γ^+^	γ^−^
		/mJ·m^−2^	/mJ·m^−2^	/mJ·m^−2^	/mJ·m^−2^
Distilled water (H_2_O)	[48]	72.8	21.8	25.5	25.5
Glycerol (C_3_H_8_O)	[48]	64.0	34	3.92	57.4
Diiodomethane (CH_2_I_2_)	[48]	50.8	50.8	0	0

**Table 3:** Surface roughness parameters (R_a_, R_c_, R_sm_) of the surface-textured HAp ceramics classified into macro- and micro-surface texturing determined by confocal microscopy. In the case of the macro-textured HAp ceramics, the roughness values describe the periodic waviness of the texturing types.

Texturing Type	Surface Type/Surface Treatment	Roughness			Surface Coefficient S_A_ ***	
		R_a_	R_c_	R_sm_	Experimental	Theory
		/µm	/µm	/µm	(real surface)	(CAD model)
**Macrotexturing (micro molding)**		*	*	*	*	
A	Non-textured	0.98	4.38	142.92	1.3	1.0
B	Linear grooves	190.47	463.67	470.66	3.3	2.8
C	Cylindric pits	203.96	499.72	946.47	3.5	2.78
D	Linear waves	123.46	428.47	486.71	2.4	2.06
E	Gaussian hills	87.22	295.08	479.10	1.7	1.66
**Microtexturing (acid etching)**		**	**	**	**	
A	Polished (P)	0.03	0.398	55.21	1.1	1.0
A	HCl (H)	2.93	13.57	175.92	2.2	-
A	Tartaric acid (T)	9.80	34.20	182.10	1.7	-

* Analyzed cross-sectional area: 5 × 5 mm^2^ = 50 × magnification. ** Analyzed cross-sectional area: 3.3 × 0.52 mm^2^ = 200–1000 × magnification (depending on surface roughness). *** Surface coefficient SA represents the ratio of the real surface area to the cross-sectional area.

**Table 4:** Surface characteristics of the micro- and nano-textured HAp ceramics: Calculated total surface energy, its disperse and polar fractions and the theoretical adhesion energy to PCL according to LWAB theory.

Sample *	Surface Energies (γ)					Theoretical Adhesion Energy (PCL)
	γ^tot^	γ^LW^	γ^AB^	γ^+^	γ^−^	$W_{\mathrm{PCL}}^{A}$
	/mJ·m^−2^	/mJ·m^−2^	/mJ·m^−2^	/mJ·m^−2^	/mJ·m^−2^	/mJ·m^−2^
Polished HAp (P)	49.3	39.3	10.0	0.4	66.9	85.3
Reference HAp [64]	55.8	45.5	10.3	0.5	53.2	*-*
Silanized (S)	46.6	42.2	4.4	0.1	33.7	84.9
HCl-etched (H)	78.8	42.9	35.9	4.4	73.2	99.7
Tartaric acid-etched (T)	90.5	45.6	44.9	5.7	89.3	104.7
T+S	82.2	44.6	37.6	4.8	73.3	101.9
PCL	38.7	36.4	2.3	0.1	13.0	-
Reference PCL [63]	26.5	24.4	2.1	0.2	5.2	*-*

* Before the application of all surface treatments, the ceramic samples were polished with a finish of 1 µm diamond paste.
